# On the Preparation of Prescriptions by Druggists

**Published:** 1810-01

**Authors:** 


### *To the Editors of the Medical and Physical Journal.*

GENTLEMEN,

I Have always been in the habit of considering Medical Practitioners as men of benevolent minds, and acting in general upon liberal principles, worthy of their erudition and science; but, if I mistake not, there is *one* exception to this sentiment to be observed, which I beg leave, thro' the medium of your useful Miscellany, to point out.

It is well understood that most physicians are in the habit of prescribing for patients at their own houses, as well as visiting others abroad. And, whether their patients are engaged with a family apothecary or not, they are sometimes instructed to carry the prescription to a certain druggist's shop (for there are physicians who have their favourite houses) in order to be prepared. It is not for me here to hazard an opinion as to their motives for so doing; but surely, it is not out of place to point out the propriety of inquiring first, if there is a medical man who is employed in the family, and if so, to intrust their prescriptions to his care, in preference to the hackneyed custom of sending them to a common druggist.

Further, in case the patients attended to, are *not* provided with a family attendant, it is very well known to the physicians who constitute the Royal College, that there are apothecaries, residing in various parts of the metropolis, who are furnished with medicines from the first house in the kingdom (Apothecaries' Hall), the genuineness and purity of which are universally acknowledged; yet, strange to tell! an illiterate druggist, who has not erudition enough to construe the prescriptions sent him, nor the medicines in sufficient purity (in the estimation of some) to be confided in, as it respects critical cases, is employed to prepare them.

Nor

Nor is this the conduct of the higher branches of the profsesion only, for surgeons, also of late years, are become home physicians, and some of them adopt the same plan. How far they infringe on the province of the regular physician in this, I presume not to say; it is enough for me to observe, that many of them have their favourite compounding shops, and the prescriptions must be carried thither, whether they have any previous connection with a medical practitioner or not.

Does not this way of preceeding deviate somewhat from the *liberality* which in general characterizes the medical profession? And, does it not place the druggist in a department not exactly his *own*, when he is employed minutely to prepare medical prescriptions, instead of vending wholesale and retail the articles peculiar to his station? I will only further add, that, if accuracy of judgement, *genuineness* of medicines, &c. be judged of any importance in the preparation of medical prescription, it scarcely needs be mentioned where they are most likely to be combined.

I am, &c.

OBSERVATOR.

*Nov.* 24, 1809.

